# First hybrid complete genome of *Aeromonas veronii* reveals chromosome-mediated novel structural variant *mcr-3.30* from a human clinical sample

**DOI:** 10.1099/acmi.0.000103

**Published:** 2020-02-17

**Authors:** Naveen Kumar Devanga Ragupathi, Dhiviya Prabaa Muthuirulandi Sethuvel, Shalini Anandan, Dhivya Murugan, Kalaiarasi Asokan, Ramya Gajaraj Neethi Mohan, Karthick Vasudevan, Thirumal Kumar D, George Priya Doss C, Balaji Veeraraghavan

**Affiliations:** ^1^​ Department of Clinical Microbiology, Christian Medical College, Vellore – 632004, India; ^2^​ School of Bio Sciences and Technology, Vellore Institute of Technology, Vellore – 632014, India

**Keywords:** *mcr-3*, colistin, *Aeromonas*, IS*As18*, *bla*OXA-12, *bla*CEPH-A3

## Abstract

Recent findings demonstrate the origin of the plasmid-mediated colistin resistance gene *mcr-3* from aeromonads. The present study aimed to screen for plasmid-mediated colistin resistance among 30 clinical multidrug-resistant (MDR) *Aeromonas* spp. PCR was used to screen for the presence of *mcr-1*, *mcr-2*, *mcr-3* and *mcr-4*, which revealed *mcr-3* in a colistin-susceptible isolate (FC951). All other isolates were negative for *mcr*. Sequencing of FC951 revealed that the *mcr-3* (*mcr-3.30*) identified was different from previously reported variants and had 95.62 and 95.28 % nucleotide similarity with *mcr-3.3* and *mcr-3.10*. Hybrid assembly using IonTorrent and MinION reads revealed structural genetic information for *mcr-3.30* with an insertion of IS*As18* within the gene. Due to this, *mcr-3.30* was non-expressive, which makes FC951 susceptible to colistin. Further, *in silico* sequence and protein structural analysis confirmed the new variant. To the best of our knowledge, this is the first report on a novel *mcr-3* variant from India. The significant role of *mcr*-like genes in different *Aeromonas* species remains unknown and requires additional investigation to obtains insights into the mechanism of colistin resistance.

## Introduction

*Aeromonas* spp. are ubiquitous and are known to cause gastroenteritis, wound infections and septicaemia; they are commonly known as ‘jacks of all trades’. Aeromonads are universally resistant to the penicillin group of antibiotics (penicillin, ampicillin, carbenicillin and ticarcillin) and are generally susceptible to tetracyclines and quinolones [1]. Recently, increasing resistance to third-generation cephalosporins and carbapenems has been reported [2, 3].

The recent discovery of plasmid-mediated colistin resistance genes (*mcr*) has attracted global attention. A study reported that the *mcr-3* identified in *Escherichia coli* is similar to the gene present in *Aeromonas* species and suggested that it might have originated from aeromonads [4]. It should be noted that most *Aeromonas* species are susceptible to colistin, whereas *Aeromonas jandaei* and *Aeromonas hydrophila* have been reported to be intrinsically resistant to polymyxins [4]. However, the role of *mcr*-like genes in different *Aeromonas* species is not clearly understood and requires further investigation.

This study examined the presence of plasmid-mediated colistin resistance among *Aeromonas* spp. using PCR and structural analysis with next-generation sequencing. The nucleotide sequences obtained using experimental methods were translated into a protein sequence, and the 3D structure was modelled using *in silico* approaches to understand the structural changes in the different variants of *mcr*.

## Methods

### Isolates and identification

A total of 30 *Aeromonas* spp. isolated from stool specimens collected from January to December 2017 from symptomatic patients attending the Christian Medical College, Vellore were included in the study. Isolation and identification of the genus and species were carried out using a standard culture and biochemical tests [5].

### Antimicrobial susceptibility testing (AST)

#### Disc diffusion

AST testing was carried out using the Kirby–Bauer disk diffusion method. The antimicrobial agents tested were trimethoprim/sulfamethoxazole (1.25/23.75 µg), tetracycline (30 µg), ciprofloxacin (5 µg), cefotaxime (30 µg), imipenem (10 µg) and meropenem (10 µg) (Oxoid, UK). Quality control (QC) strains (* Klebsiella pneumoniae* ATCC 700603, *Pseudomonas aeruginosa* ATCC 27853 and *E. coli* ATCC 25922) were included in all batches, as recommended by the Clinical and Laboratory Standards Institute (CLSI-M45) [6].

### Minimum inhibitory concentration (MIC)

The colistin MIC was determined for the studied isolates by broth microdilution and interpreted using the CLSI 2017 breakpoint recommendation [7]. *mcr-1*-positive *E. coli* with the expected range 4–8 µg ml^−1^, *E. coli* ATCC 25922 (0.25–2 µg ml^−1^) and *P. aeruginosa* ATCC 27853 (0.5–4 µg ml^−1^) were used as QC) strains for colistin MIC determination.

### Screening of *mcr* genes by PCR

The presence of *mcr-1*, *mcr-2*, *mcr-3* and *mcr-4* encoding for plasmid-mediated colistin resistance was screened by PCR as described previously [4, 6–8].

### Next-generation sequencing

The isolate that was positive for *mcr* was selected for next-generation sequencing to analyse colistin resistance determinants and other genetic factors. A QIAamp DNA Mini kit (Qiagen, Hilden, Germany) was used for genomic DNA extraction. Whole-genome sequencing (WGS) of the isolate was performed with 400 bp read chemistry using an IonTorrent Personal Genome Machine (PGM) (Life Technologies, Carlsbad, CA, USA) as per the manufacturer’s instructions. Data were assembled *de novo* using Assembler SPAdes v.5.0.0.0 embedded in Torrent Suite Server v.5.0.3. Sequence annotation was performed using PATRIC, the bacterial bioinformatics database and analysis resource (http://www.patricbrc.org), and the National Center for Biotechnology Information’s (NCBI’s) Prokaryotic Genomes Automatic Annotation Pipeline (PGAAP, http://www.ncbi.nlm.nih.gov/genomes/static/Pipeline.html).

The CGE server (http://www.cbs.dtu.dk/services) and PATRIC [8] were employed for downstream analysis. ResFinder 2.1 (https://cge.cbs.dtu.dk//services/ResFinder/) was used to analyse the resistance gene profile [9]. Antimicrobial resistance genes were also screened using the Antibiotic Resistance Genes Database (ARDB) and the Comprehensive Antibiotic Resistance Database (CARD) through PATRIC. PlasmidFinder 1.3 (https://cge.cbs.dtu.dk//services/PlasmidFinder/) was used to screen for the presence of plasmids [10]. The clustered regularly interspaced short palindromic repeats (CRISPR) and Cas genes was identified from the genome using the CRISPRFinder tool [11]. The MultiLocus Sequence Typing (MLST) 1.8 tool was employed for sequence type analysis (https://cge.cbs.dtu.dk//services/MLST/) [12]. The genome was screened for insertion sequence elements using ISFinder (https://www-is.biotoul.fr/blast.php) [13].

### MinION Oxford Nanopore sequencing

DNA library preparation and sequencing was prepared using SQK-LSK108 Kit R9 version (Oxford Nanopore Technologies, Oxford, UK) using a 1D sequencing method according to the manufacturer’s protocol. Sequencing was performed using the FLO-MIN106 R9 flow cell in the MinION Mk 1B sequencer. MinKNOW software v 1.15.1 (Oxford Nanopore Technologies, Oxford, UK) was employed in a Windows platform to perform sequencing and raw data (fast5 files) were obtained.

### MinION sequence analysis

The fast5 files were generated from MinION sequencing and the reads were base-called with Albacore 2.0.1 (https://nanoporetech.com/about-us/news/new-basecaller-now-performs-raw-basecalling-improved-sequencing-accuracy). Furthermore, the adapters were trimmed using Porechop (https://github.com/rrwick/Porechop). Canu 1.7 [14] was used for MinION error correction and assembly with a genome size of 5.0 m as input. After *de novo* assembly, the contigs were polished with Nanopolish 0.10.1 (https://github.com/jts/nanopolish).

### Hybrid assembly using IonTorrent and MinION reads

To increase the accuracy and completeness of he genome, a hybrid assembly using both IonTorrent and MinION reads with Unicycler (v0.4.6) was performed [15]. By default, Unicycler utilizes SPAdes [16] to assemble the short reads with different k-mers and filter out the low-depth regions. Subsequently, it trims and generates the short-read assembly graph. In addition, it uses Miniasm [17] and Racon [18] to assemble the MinION long reads and further the reads were bridged to determine all the genome repeats and produce complete genome assembly. The short reads were also polished with multiple rounds of Pilon [19] to reduce the base-level errors. After assembly, the assembly statistics and average nucleotide identity of different assemblies were evaluated using the Quast [20] and OrthoANI 0.93.1 tools [21], respectively.

### *In silico* sequence analysis

The *mcr* nucleotide sequences obtained from the experimental techniques were translated to a protein sequence using the online Expasy Translate tool (https://web.expasy.org/translate/). The percentage identity of known MCR variants in comparison with novel protein was identified using blast search. The conserved amino acid region from the closely related variants was determined using Clustal Omega.

### *In silico* structure analysis

The sequences of the MCR variant was used to model the 3D structure of the proteins. The 3D structures of the variants were modelled using Swiss-Model [22]. The translated variant sequences were given as the input for the 3D variant modelling. The rampage server was used to evaluate the quality of the modelled variants [23]. Finally, the MetaPocket server was used to predict the active pocket of the novel variant [24]. The structure visualization was performed using PyMOL.

## Results

### Antimicrobial susceptibility

The resistance profiles for the studied *Aeromonas* isolates are presented in Table 1. The colistin MIC was determined for all the isolates and this showed 30 % of the isolates were resistant to colistin. The MIC was identified to be 0.5 µg ml^−1^ (susceptible) for the isolate that was positive for *mcr-3*.

**Table 1.** Antimicrobial susceptibility of the selected *Aeromonas* spp.

Sample no.	Sample ID	Age/sex	Organism	Resistance pattern (disc diffusion)	Colistin MIC (µg ml^−1^)
1	FC3340	29M	* Aeromonas* spp.	AMP-SXT-TAX	4R
2	FC193	0M	* A. dhakensis *	AMP-IMI-MEM	≥64R
3	FC199	76M	* A. hydrophila *	AMP	1S
4	FC284	75M	* Aeromonas* spp.	AMP-IMI-MEM	16R
5	FC728	71M	* A. caviae *	AMP-TET-TAX-IMI-MEM-CIP	0.5S
6	FC729	66M	* A. caviae *	AMP-TET-SXT-TAX-IMI-MEM-CIP	0.5S
7	FC715	34F	* A. caviae *	AMP	>32R
8	FC850	27M	* A. veronii *	AMP-TET-SXT-IMI-MEM-CIP	1S
9	*FC951	55M	* A. veronii *	AMP-TET-IMI-MEM	0.5S
10	FC1239	21F	* A. dhakensis *	AMP-TAX-IMI-MEM	≥64R
11	FC1520	45F	* A. hydrophila *	AMP	>32R
12	FC1169	0M	* A. hydrophila *	AMP	2S
13	FC599	57F	* A. hydrophila *	AMP-SXT	2S
14	FC814	55F	* A. hydrophila *	AMP-IMI	>32R
15	FC2457	0M	* A. hydrophila *	AMP-TET-SXT	2S
16	FC538	58M	* A. hydrophila *	AMP-IMP	2S
17	FC771	1M	* A. caviae *	AMP-TAX-CIP	4R
18	FC578	26F	* A. caviae *	AMP	1S
19	FC377	22F	* A. hydrophila *	AMP-SXT-IMI-MEM	1S
20	FC1245	42F	* A. hydrophila *	AMP-IMI	2S
21	FC788	0M	* A. hydrophila *	AMP-TAX-IMI	8R
22	FC157	58M	* A. veronii *	AMP	0.5S
23	FC390	9F	* A. caviae *	AMP	1S
24	FC1411	5M	* A. hydrophila *	AMP-IMI	2S
25	FC523	1M	* A. hydrophila *	AMP-TET	1S
26	FC1999	54F	* A. hydrophila *	AMP-IMI-MEM	0.5S
27	FC2051	28M	* A. caviae *	AMP	1S
28	FC2019	60M	* A. caviae *	AMP	1S
29	FC906	55M	* A. hydrophila *	AMP-TET	0.5S
30	FC1435	73F	* A. hydrophila *	AMP	>32R

*Isolate sequenced: R, resistant; S, susceptible; AMP, ampicillin; TET, tetracycline; SXT, trimethoprim/sulfamethoxazole; CIP, ciprofloxacin; TAX, cefotaxime; IMI, imipenem; MEM, meropenem.

### Screening of *mcr* genes

Of the 30 isolates screened for *mcr-1*, *mcr-2*, *mcr-3* and *mcr-4*, only one isolate (FC951) was positive for *mcr-3*.

## Next-generation sequencing

The *A. veronii* (FC951) hat was positive for *mcr-3* by PCR was sequenced using IonTorrent PGM. Analysis of *mcr-3* revealed only 95.6 % identity against the reference sequences in the database (henceforth termed *mcr-3.30*). Further, ISFinder revealed that IS*As18* belongs to the IS*4* family next to *eptA,* aka *mcr-3*.

**Fig. 1.**
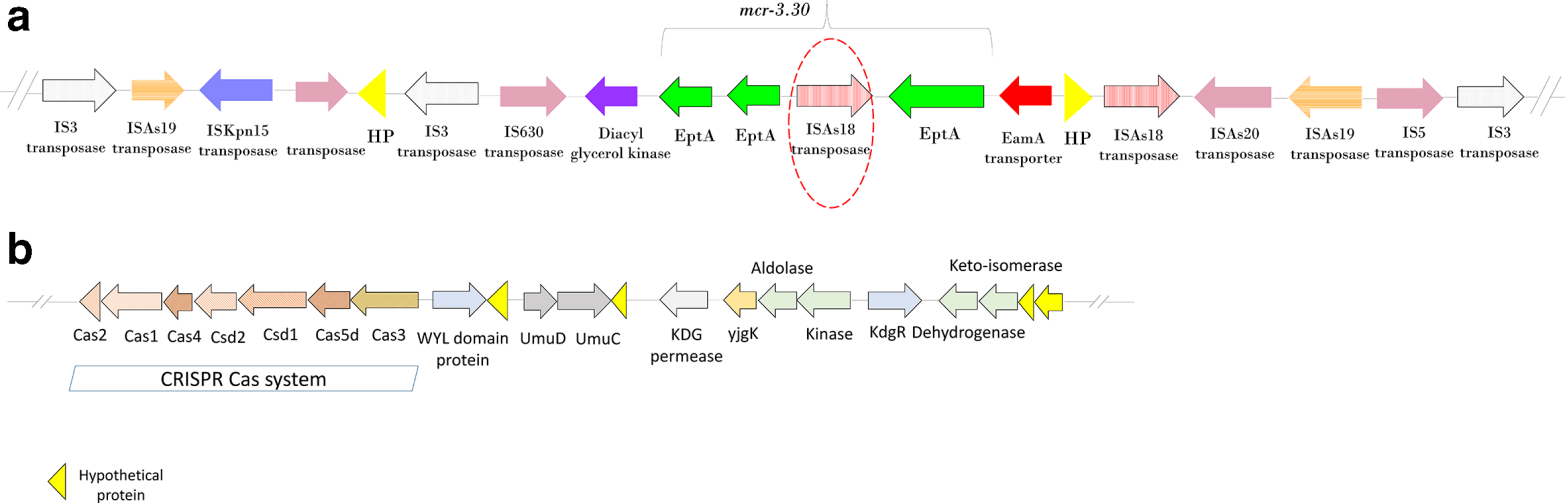
(a) Genetic environment of *mcr-3.30* with an insertion of IS*As18* transposase (1141 bp) leading to disruption of *mcr-3.30* function. (b) CRISPR Cas system identified in FC951 and arrangement of Cas genes.

However, the *mcr-3.30* was split in to two contigs (IonTorrent). Generally, the IonTorrent assembly is highly accurate, but the assembly had too many fragments. The long-read sequencing of FC951 using MinION resulted in a complete genome (a chromosome and a plasmid), but with errors. Hybrid assembly using IonTorrent and MinION reads in Unicycler resulted a complete chromosomal contig for FC951 with increased accuracy and few errors. Interestingly, analysis of the complete genome revealed *mcr-3.30* integrated in the chromosome along with the insertion of IS*As18* within *mcr-3.30*. The structure of the genetic environment of *mcr-3.30* is shown in Fig. 1a.

The Quast analysis showed the N50 and N75 value of the hybrid assembly to be 4 660 178, which is approximately 96 % of the total assembly length. In addition, ANI is calculated using different assembly methods. It is evident that the closeness between IonTorrent and hybrid assembly is about 99.92 %, which represents the high accuracy of the hybrid assembly. The hybrid assembly generated a 4.66 Mbp chromosome length single contig. In contrast, the IonTorrent-only assembly produced an assembly with more than 300 contigs and only 139 contigs >1000 bp. It was clear that hybrid assembly has its own advantages, with improved accuracy and a reduced error rate with genome completeness compared to MinION-only or IonTorrent-only assembly.

Moreover, annotation of the extra-chromosomal sequences from MinION could not be designated as a complete plasmid, although it showed 21 % similarity with a previously reported *Xanthomonas citri* plasmid (CP020883.1).

Further analysis of resistance genes using ResFinder revealed the presence of *bla*_OXA12_ and *bla*_CEPH-A3_ genes. A CRISPR Cas system was identified in FC951 and the arrangements of the genes were as shown in Fig. 1b. Notably, the sequence type of FC951 was identified to be novel, ST-515.

This complete genome project has been deposited at GenBank under the accession number CP032839 (plasmid accession number CP032840).

### *In silico* sequence analysis

The *mcr-3* nucleotide and protein sequence identified in this study was compared with the previously reported variants *mcr-3.1*–*mcr-3.10* using blast search (KY924928.1, NMWW01000143.1, MF495680, NQCO01000074.1, MF489760, MF598076.1, MF598077.1, MF598078.1, MF598080.1 and MG214531) and identified to be a novel variant (*mcr-3.30*). The nucleotide percentage identity matrix for the *mcr* gene variants was as given in Table 2. From the analysis, *mcr-3.3* and *mcr-3.10* were found to be highly identical (95.62 and 95.28 %, respectively. The most conserved amino acid region among the three variants (*mcr-3.3*, *mcr-3.10* and *mcr-3.30*) was identified using Clustal Omega. The region of amino acids from LEU-359 to ILE-427 was found to be the largest conserved sequence among them.

**Table 2.** Percentage identity matrix of *mcr-3* variants in comparison with FC951 *mcr-3.30*

	*mcr-3.30* (FC951)	*mcr-3.6*	*mcr-3.3*	*mcr-3.8*	*mcr-3.7*	*mcr-3.5*	*mcr-3.4*	*mcr-3.1*	*mcr-3.2*	*mcr-3.9*	*mcr-3.10*
*mcr-3.30* (FC951)	**100**										
*mcr-3.6*	**94.07**	100									
*mcr-3.3*	**95.62**	98.15	100								
*mcr-3.8*	**95.01**	98.64	98.95	100							
*mcr-3.7*	**92.6**	94.33	94.09	93.97	100						
*mcr-3.5*	**94.07**	93.72	95.32	94.22	95.69	100					
*mcr-3.4*	**94.13**	93.72	95.32	94.22	95.82	99.75	100				
*mcr-3.1*	**94.2**	93.78	95.38	94.28	95.88	99.82	99.94	100			
*mcr-3.2*	**94.2**	93.84	95.44	94.34	95.82	99.88	99.88	99.94	100		
*mcr-3.9*	**94.54**	94.39	95.75	94.9	96.62	97.72	97.85	97.91	97.85	100	
*mcr-3.10*	**95.28**	94.7	96.55	95.33	96.56	98.65	98.77	98.83	98.77	99.08	100

### *In silico* structure analysis

**Fig. 2.**
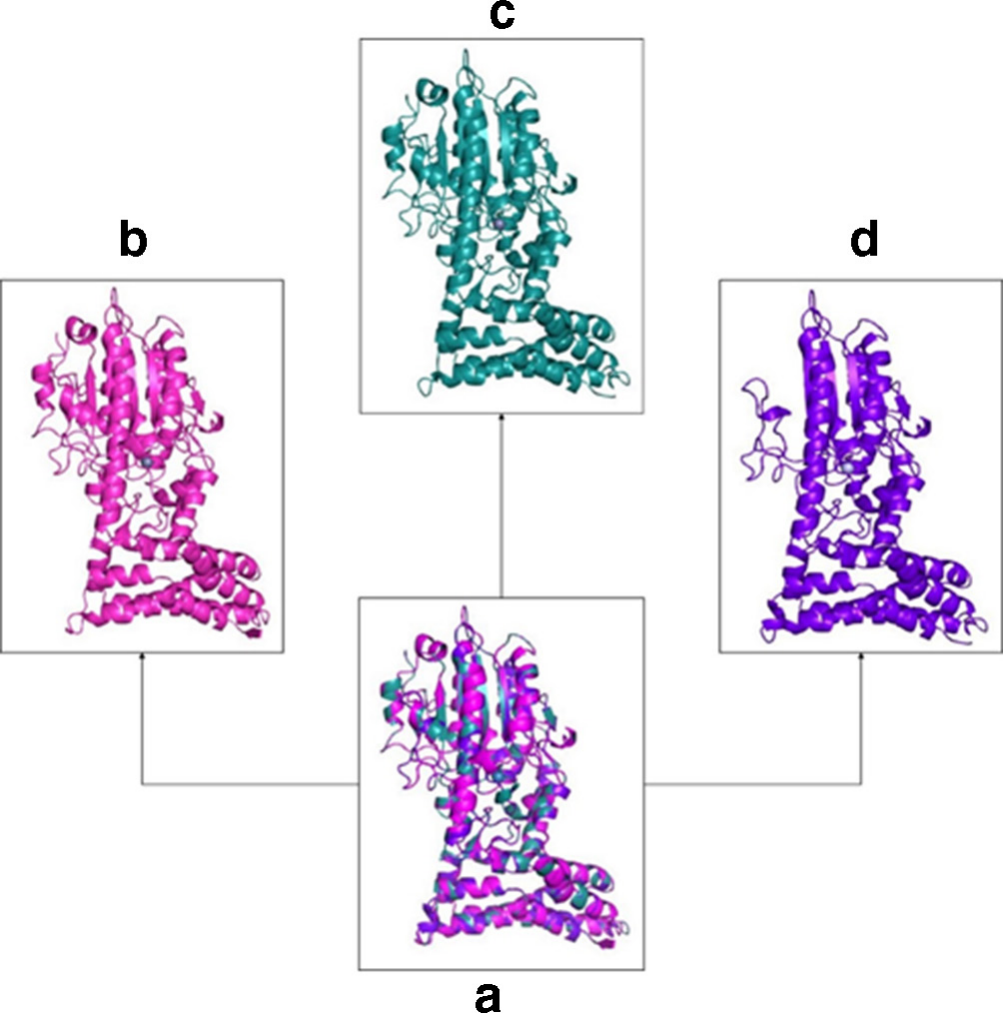
(a) Superimposed structural evaluation of MCR variant 3.30 against the closely related MCR variant 3.3 and MCR variant 3.10, (b) 3D structure of MCR variant 3.3, (c) 3D structure of MCR variant 3.10 and (d) 3D structure of MCR variant 3.30.

The 3D structures were modelled using the sequences for the variants *mcr-3.1*–*mcr-3.10* using the Swiss-Model server. The template used and its respective PDB ID, coverage range and coverage identity are tabulated in Table 3. Further, the quality of the modelled variants was evaluated using the rampage server. The percentage of the amino acids in the favoured region, the percentage of amino acids in the allowed region and the percentage of amino acids in the outlier region are tabulated in Table 3. Superimposed structural evaluation of MCR variant 3.30 against the closely related MCR variant 3.3 and MCR variant 3.10 was visualized using PyMOL (Fig. 2). In addition, LEU53, ILE164, ALA57, TYR175, GLN186, ILE189, VAL176, GLY179, ALA192, PHE32, LEU50, VAL61, ARG180, VAL178, ASN182, LEU185, PHE46, LEU36, SER183, VAL29, GLY28, PRO51, LEU54, ASN25, ALA21, TRP26, GLU188, LEU24, LEU58, PRO191, ASN193, VAL195, PHE65 and ASN196 were identified as the active site of the MCR-3.30 variant using the Meta Pocket server (Fig. S1, available in the online version of this article).

**Table 3.** List of parameters considered for modelling the variants using Swiss-Model and Ramachandran plot evaluation of the structures using rampage

Variants of *mcr*	Template PDB	Coverage range	Coverage	Identity	% of amino acids in the favoured region	% of amino acids in the allowed region	% of amino acids in the outlier region
3.1	5FGN	6–544	0.98	37.55	94.2 %	4.9 %	0.9 %
3.2	5FGN	6–545	0.98	37.55	94.2 %	4.9 %	0.9 %
3.3	5FGN	1–546	0.99	35.82	94.0 %	5.1 %	0.9 %
3.4	5FGN	6–544	0.98	37.55	94.4 %	4.7 %	0.9 %
3.5	5FGN	6–546	0.97	40.27	95.9 %	3.6 %	0.6 %
3.6	5FGN	6–546	0.97	39.69	95.7 %	3.6 %	0.8 %
3.7	5FGN	6–546	0.97	40.08	95.1 %	4.1 %	0.8 %
3.8	5FGN	6–546	0.97	39.69	95.7 %	3.6 %	80.0 %
3.9	5FGN	6–546	0.97	40.08	95.3 %	4.0 %	80.0 %
3.10	5FGN	6–544	0.97	37.74	94.6 %	4.5 %	90.0 %
3.30	5FGN	6–469	0.98	36.24	93.7 %	4.6 %	1.7 %

## Discussion

Resistance to colistin is mainly mediated via alteration of the lipopolysaccharides (LPS) of the bacterial outer membrane. The alterations include mutations in lipid A-modifying genes. The most commonly reported mutations are in the *mgrB* gene and are therefore not transferable through horizontal gene transfer [25]. However, in 2015, the first plasmid-mediated colistin resistance gene (*mcr-1*) was reported [26], which belongs to the phosphoethanolamine transferase enzyme family (*Ept*A). *mcr-1* was identified in *E. coli* from human patients and animals in China. In 2016, another study reported the mobilizable colistin resistance gene, *mcr-2,* from porcine and bovine *E. coli* isolates in Belgium [27]. Further, *mcr-3* and *mcr-4* were identified in *E. coli, Klebsiella* spp. and *Salmonella* spp. [4, 28]. Recently, *mcr-5* was identified in *Salmonella enterica* subsp. enterica serovar Paratyphi B isolated from poultry in Denmark [29].

Several mobile colistin resistance genes have been identified, but only *mcr-1* and *mcr-3* have been reported with a high number of variants in GenBank database. A recent study highlighted the importance of a third mobile colistin resistance gene, *mcr-3* in *Aeromonas salmonicida *, due to its resemblance to various other phosphoethanolamine transferases in *Enterobacteriaceae* and also suggested that this resistance gene might have already been widely disseminated [4]. Here we discuss a novel variant of *mcr-3* identified in *A. veronii* isolated from a clinical specimen.

The novel *mcr-3* variant identified in this study exhibited ≤95 % nucleotide sequence similarity to all other previously reported *mcr*-3 variants, and is henceforth named *mcr-3.30. In silico* protein sequence comparison revealed the novelty of MCR-3.30. The superimposed protein structure comparison with MCR-3.3 and MCR-3.10 further confirmed the MCR-3.30 variant. Major structural changes were observed in domain 2. Similar comparisons of MCR-3 and MCR-1 protein structures were previously reported by [4]. Knowledge of the 3D structure of proteins is now in great demand in the field of computer-aided drug discovery (CADD). It helps researchers in identifying new drugs. In this study, we have used homology modelling technique to model the 3D structures of MCR variants.

*mcr-3* was first identified in a 261 kb IncHI2-type plasmid pWJ1 from *E. coli* [4]. However, initially, known plasmid replicons were not identified in FC951 harbouring *mcr-3.30* via PlasmidFinder. Later, sequence assembly and alignment with pWJ1 revealed an Inc-W like replicase gene in the extrachromosomal region, along with TraI and TrbN genes responsible for multi-functional conjugation and conjugal transfer proteins.

There had been a previous report on the chromosome integration of the *mcr-3* variant in *A. veronii* [30]. In this study, a major insertion of IS*As18* (1141 bp) belonging to the IS*4* family of insertional elements was found within *mcr-3.30*. IS*As18* had previously only been reported in *A. salmonicida* as a transposase [31]. The entire *mcr-3.30* region, including the insertion, was flanked by *eamA* and *dgkA*. These genes encode a metabolite transporter and a diacylglycerol kinase, respectively [32]. The *mcr-3.30* genetic environment also had other insertional elements, such as IS*As19*, IS*As20*, IS*630*, IS*Kpn15*, IS*5* and IS*3* transposase.

However, *mcr-3.30* disruption caused by the insertion of IS*As18* has rendered it non-expressive. Accordingly, the isolate FC951, in spite of harbouring *mcr-3.30,* was phenotypically susceptible to colistin. Similarly, a previous study by Pham Thanh *et al*. identified a deactivated *mcr-1* due to the disruption of the gene by a 22 bp duplication in colistin-susceptible *Shigella sonnei* [33]. The gene was found to be reactivated by conjugation experiments. resulting in a colistin-resistant phenotype. Another study by Liassine *et al*. reported *mcr-1* in susceptible *E. coli* with an unknown cause of susceptibility [34]. A recent study showed the presence of *mcr-1* in susceptible *E. coli* due to the insertion of 1329 bp transposon IS10R and found that this could not be reactivated. As shown from these studies, the inactivated gene can be restored upon colistin exposure, particularly in settings where colistin use is high. This phenomenon emphasizes the importance of phenotypic confirmation despite detection of the gene in molecular screening [33, 35]. In contrast, the isolates of the present study that were resistant to colistin by MIC were negative for screened *mcr*; this could be due to other chromosomal mechanisms that need to be explored.

There are no reports of *mcr* variants other than *mcr-1* from India. Various Indian centres have reported colistin-resistant strains in hospitalized patients. So far, there are only published reports of colistin resistance in *Enterobacteriaceae* and non-fermenters such as *E. coli *, *K. pneumoniae* and *A. baumannii* from India.

Mutation in the *mgr*B gene is the most common resistance mechanism in *K. pneumoniae* and mutation in the *lpx*A/D, *lps*B and *pmr*B genes is responsible for resistance in *A. baumannii*. On the other hand, the presence of plasmid-mediated *mcr* confers resistance in *E. coli* [25, 36, 37]

Apart from the colistin resistance gene, the genome analysis revealed the presence of other resistance genes, such as *bla*_OXA-12_, belonging to class D beta-lactamase in FC951, which confers resistance to ampicillin and is known to be naturally produced by *Aeromonas jandaei* and has strong activity against oxacillin [38]. The isolate also harboured *bla*_CEPH-A3_, which is the most common metallo-beta-lactamase (MBL) produced by *Aeromonas* species responsible for carbapenem resistance.

### Conclusion

To the best of our knowledge, this is the first report on a novel *mcr-3* variant (*mcr-3.30*) at the structural level, in comparison with the known variants (MCR-3.3–MCR-3.10). The *mcr-3.30* identified in this study is non-functional for colistin resistance due to the insertion of IS*As18* within the gene. Further, this is the first complete genome sequence of *A. veronii* from India, and the first hybrid genome of *A. veronii* globally. These findings support extended screening of known and further exploration of unknown colistin resistance mechanisms in this pathogen as well as in other Gram-negative pathogens.

## Supplementary Data

Supplementary material 1

## References

[1] Batra P, Mathur P, Misra MC (2016). *Aeromonas spp*.: an emerging nosocomial pathogen. J Lab Physicians.

[2] Bhaskar M, Dinoop KP, Mandal J (2015). Characterization of ceftriaxone-resistant *Aeromonas spp*. isolates from stool samples of both children and adults in southern India. J Health Popul Nutr.

[3] Anandan S, Gopi R, Devanga Ragupathi NK, Muthuirulandi Sethuvel DP, Gunasekaran P (2017). First report of bla_OXA-181_-mediated carbapenem resistance in *Aeromonas caviae* in association with pKP3-A: Threat for rapid dissemination. J Glob Antimicrob Resist.

[4] Yin W, Li H, Shen Y, Liu Z, Wang S (2017). Novel Plasmid-Mediated Colistin Resistance Gene *mcr-3* in *Escherichia coli*. MBio.

[5] Abbott SL, Cheung WKW, Janda JM (2003). The genus *Aeromonas*: biochemical characteristics, atypical reactions, and phenotypic identification schemes. J Clin Microbiol.

[6] Clinical and Laboratory Standards Institute (CLSI 2016 Methods for antimicrobial dilution and disk susceptibility testing of infrequently isolated or fastidious bacteria. Document M45.

[7] Clinical and Laboratory Standards Institute (CLSI 2017 Performance standards for antimicrobial susceptibility testing. Twenty-seventh Informational Supplement M100-S27.

[8] Wattam AR, Abraham D, Dalay O, Disz TL, Driscoll T (2014). PATRIC, the bacterial bioinformatics database and analysis resource. Nucleic Acids Res.

[9] Zankari E, Hasman H, Cosentino S, Vestergaard M, Rasmussen S (2012). Identification of acquired antimicrobial resistance genes. Journal of Antimicrobial Chemotherapy.

[10] Carattoli A, Zankari E, Garcìa-Fernandez A, Larsen MV, Lund O (2014). And pMLST: in silico detection and typing of plasmids. Antimicrob Agents Chemother.

[11] Grissa I, Vergnaud G, Pourcel C (2007). CRISPRFinder: a web tool to identify clustered regularly interspaced short palindromic repeats. Nucleic Acids Res.

[12] Larsen MV, Cosentino S, Rasmussen S, Friis C, Hasman H (2012). Multilocus sequence typing of total-genome-sequenced bacteria. J Clin Microbiol.

[13] Siguier P, Pérochon J, Lestrade L, Mahillon J, Chandler M (2006). ISfinder: the reference centre for bacterial insertion sequences. Nucleic Acids Res.

[14] Koren S, Walenz BP, Berlin K, Miller JR, Bergman NH (2017). Canu: scalable and accurate long-read assembly via adaptive *k* -mer weighting and repeat separation. Genome Res.

[15] Wick RR, Judd LM, Gorrie CL, Holt KE (2017). Unicycler: resolving bacterial genome assemblies from short and long sequencing reads. PLoS Comput Biol.

[16] Bankevich A, Nurk S, Antipov D, Gurevich AA, Dvorkin M (2012). SPAdes: a new genome assembly algorithm and its applications to single-cell sequencing. J Comput Biol.

[17] XP L, Fang LX, Song JQ, Xia J, Huo W (2016). Clonal spread of mcr-1 in PMQR-carrying ST34 Salmonella isolates from animals in China. Sci Rep.

[18] Vaser R, Sović I, Nagarajan N, Šikić M (2017). Fast and accurate de novo genome assembly from long uncorrected reads. Genome Res.

[19] Walker BJ, Abeel T, Shea T, Priest M, Abouelliel A (2014). Pilon: an integrated tool for comprehensive microbial variant detection and genome assembly improvement. PLoS One.

[20] Gurevich A, Saveliev V, Vyahhi N, Tesler G (2013). QUAST: quality assessment tool for genome assemblies. Bioinformatics.

[21] Lee I, Ouk Kim Y, Park S-C, Chun J (2016). OrthoANI: an improved algorithm and software for calculating average nucleotide identity. Int J Syst Evol Microbiol.

[22] Biasini M, Bienert S, Waterhouse A, Arnold K, Studer G (2014). SWISS-MODEL: modelling protein tertiary and quaternary structure using evolutionary information. Nucleic Acids Res.

[23] Lovell SC, Davis IW, Arendall WB, de Bakker PI, Word JM (2003). Structure validation by Calpha geometry: phi, psi and Cbeta deviation. Proteins.

[24] Zhang Z, Li Y, Lin B, Schroeder M, Huang B (2011). Identification of cavities on protein surface using multiple computational approaches for drug binding site prediction. Bioinformatics.

[25] Pragasam AK, Shankar C, Veeraraghavan B, Biswas I, Nabarro LEB (2017). Molecular mechanisms of colistin resistance in Klebsiella pneumoniae causing bacteremia from India—A first report. Front Microbiol.

[26] Liu Y-Y, Wang Y, Walsh TR, Yi L-X, Zhang R (2016). Emergence of plasmid-mediated colistin resistance mechanism MCR-1 in animals and human beings in China: a microbiological and molecular biological study. Lancet Infect Dis.

[27] Xavier BB, Lammens C, Ruhal R, Kumar-Singh S, Butaye P (2001). Identification of a novel plasmid-mediated colistin-resistance gene, mcr-2, in *Escherichia coli*, Belgium, June 2016. Eurosurveillance.

[28] Carattoli A, Villa L, Feudi C, Curcio L, Orsini S (2017). Novel plasmid-mediated colistin resistance *mcr-4 g* ene in *Salmonella* and *Escherichia coli*, Italy 2013, Spain and Belgium, 2015 to 2016. Eurosurveillance.

[29] Borowiak M, Fischer J, Hammerl JA, Hendriksen RS, Szabo I (2017). Identification of a novel transposon-associated phosphoethanolamine transferase gene, mcr-5, conferring colistin resistance in d-tartrate fermenting Salmonella enterica subsp. enterica serovar paratyphi B. J Antimicrob Agents Chemother.

[30] Ling Z, Yin W, Li H, Zhang Q, Wang X (2017). Chromosome-Mediated *mcr-3* Variants in *Aeromonas veronii* from Chicken Meat. Antimicrob Agents Chemother.

[31] Pfeiffer F, Zamora-Lagos M-A, Blettinger M, Yeroslaviz A, Dahl A (2018). The complete and fully assembled genome sequence of *Aeromonas salmonicida* subsp. pectinolytica and its comparative analysis with other Aeromonas species: investigation of the mobilome in environmental and pathogenic strains. BMC Genomics.

[32] Kieffer N, Nordmann P, Moreno AM, Zanolli Moreno L, Chaby R (2018). Genetic and Functional Characterization of an MCR-3-Like Enzyme-Producing *Escherichia coli* Isolate Recovered from Swine in Brazil. Antimicrob Agents Chemother.

[33] Pham Thanh D, Thanh Tuyen H (2016). Nguyen THI Nguyen T, Chung the H, wick Rr, Thwaites Ge, baker S, Holt Ke. inducible colistin resistance via a disrupted plasmid-borne mcr-1 gene in a 2008 Vietnamese Shigella sonnei isolate. J Antimicrob Agents Chemother.

[34] Liassine N, Assouvie L, Descombes M-C, Tendon VD, Kieffer N (2016). Very low prevalence of MCR-1/MCR-2 plasmid-mediated colistin resistance in urinary tract Enterobacteriaceae in Switzerland. Int J Infect Dis.

[35] Terveer EM, Nijhuis RHT, Crobach MJT, Knetsch CW, Veldkamp KE (2017). Prevalence of colistin resistance gene (mcr-1) containing Enterobacteriaceae in feces of patients attending a tertiary care hospital and detection of a mcr-1 containing, colistin susceptible E. coli. PLoS One.

[36] Ghafur A, Shankar C, GnanaSoundari P, Venkatesan M, Mani D (2019). Detection of chromosomal and plasmid-mediated mechanisms of colistin resistance in Escherichia coli and Klebsiella pneumoniae from Indian food samples. J Glob Antimicrob Resist.

[37] Vijayakumar S, S BA, Kanthan K, Veeraraghavan B (2018). Whole-Genome shotgun sequences of seven colistin-resistant Acinetobacter baumannii isolates from bacteraemia. J Glob Antimicrob Resist.

[38] Poirel L, Naas T, Nordmann P (2010). Diversity, epidemiology, and genetics of class D beta-lactamases. Antimicrob Agents Chemother.

