# Supplementary material for: First hybrid complete genome of Aeromonas veronii reveals chromosome-mediated novel structural variant mcr-3.30 from a human clinical sample
Source: Access Microbiol. 2020 Feb 17;2(4):acmi000103. doi: 10.1099/acmi.0.000103 (PMC7523623; doi:10.1099/acmi.0.000103)

Figure S1: LEU53, ILE164, ALA57, TYR175, GLN186, ILE189, VAL176, GLY179, ALA192, PHE32, LEU50, VAL61, ARG180, VAL178, ASN182, LEU185, PHE46, LEU36, SER183, VAL29, GLY28, PRO51, LEU54, ASN25, ALA21, TRP26, GLU188, LEU24, LEU58, PRO191, ASN193, VAL195, PHE65, and ASN196 amino acids were identified as active sites of MCR-3.30 using Meta Pocket server.

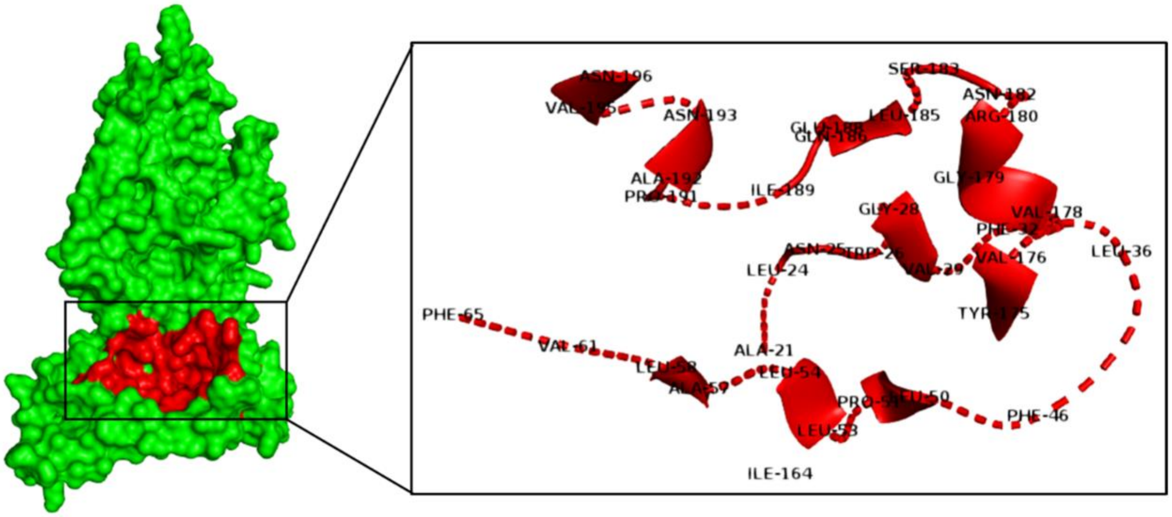

Supplement: Supplementary material 1 [file acmi-2-103-s001.pdf]
